# A Study to Explore the Suitability of LiNi_0.8_Co_0.15_Al_0.05_O_2_/Silicon@Graphite Cells for High-Power Lithium-Ion Batteries

**DOI:** 10.3390/ijms221910331

**Published:** 2021-09-25

**Authors:** Marta Cabello, Emanuele Gucciardi, Guillermo Liendo, Leire Caizán-Juananera, Daniel Carriazo, Aitor Villaverde

**Affiliations:** 1 Centre for Cooperative Research on Alternative Energies (CIC energiGUNE), Basque Research and Technology Alliance (BRTA), Alava Technology Park, 01510 Vitoria-Gasteiz, Spain; egucciardi@cicenergigune.com (E.G.); gliendo@cicenergigune.com (G.L.); leire.caizan@gmail.com (L.C.-J.); dcarriazo@cicenergigune.com (D.C.); avillaverde@cicenergigune.com (A.V.); 2 IKERBASQUE, Basque Foundation for Science, Calle María Díaz de Haro, 3, 48013 Bilbao, Spain

**Keywords:** silicon, graphite, composites, lithium-ion batteries, high power

## Abstract

Silicon–graphite (Si@G) anodes are receiving increasing attention because the incorporation of Si enables lithium-ion batteries to reach higher energy density. However, Si suffers from structure rupture due to huge volume changes (ca. 300%). The main challenge for silicon-based anodes is improving their long-term cyclabilities and enabling their charge at fast rates. In this work, we investigate the performance of Si@G composite anode, containing 30 wt.% Si, coupled with a LiNi_0.8_Co_0.15_Al_0.05_O_2_ (NCA) cathode in a pouch cell configuration. To the best of our knowledge, this is the first report on an NCA/Si@G pouch cell cycled at the 5C rate that delivers specific capacity values of 87 mAh g^−1^. Several techniques such as X-ray diffraction (XRD), scanning electron microscopy (SEM), electrochemical impedance spectroscopy (EIS) and gas chromatography–mass spectrometry (GC–MS) are used to elucidate whether the electrodes and electrolyte suffer irreversible damage when a high C-rate cycling regime is applied, revealing that, in this case, electrode and electrolyte degradation is negligible.

## 1. Introduction

In the last decade, Li-ion batteries (LIBs) have extended their uses from portable electronic devices to electric vehicles and stationary applications. To cover all these applications, new technologies with higher power and energy densities are being increasingly demanded. Currently, the main cathode materials that are considered by the automotive industry are limited to the LiNi_0.8_Co_0.15_Al_0.05_O_2_ (NCA) and LiNi_x_Mn_y_Co_z_O_2_ (NMC) families, as they are the only materials that meet energy density, power, lifetime and safe cyclability targets [1,2,3]. For instance, an NCA cathode is used in Tesla Model S vehicles [4]. NCA is considered a promising cathode in the Li-ion battery field due to its attractive features such as a high specific capacity of ca. 200 mAh g^−1^, structural stability given by the presence of Co [5] and thermal stability at high potentials because of the presence of electrochemically inactive Al^3+^ ions [6]. However, capacity fading and resistance increase are common problems associated with the long-term cycling of NCA cathodes as a consequence of microstructural degradation, parasitic reactions and phase transitions [7,8,9,10].

In addition to the handicaps mentioned above, cathode performance worsens when moving from half-cell to full-cell configuration. Therefore, the choice of anode material is a critical issue, as its characteristics can directly impact battery electrochemical performance. The anode should have stable capacity and coulombic efficiency values, as well as long cyclability and capacity retention. In addition, the anode redox potential needs to be maintained above 0 V in order to avoid Li plating. Even though graphite is the most common anode material in LIBs, silicon is being increasingly used to improve energy density in LIBs [11,12,13]. Silicon belongs to the group of Li-alloy anodes in which alloying and dealloying processes occur by multiple electron exchange mechanisms [14], providing high capacity values to these materials. Although Si has the highest gravimetric capacity (3579 mAh g^−1^ for Li_15_Si_4_) [15], these particles suffer from large volume changes during cycling (close to 300%) [16], resulting in particle cracking and pulverization, causing rupture of the solid electrolyte interphase (SEI) and thereby inducing an unceasing electrolyte decomposition and cell failure.

In order to mitigate the drawbacks associated with Si anodes, especially breakage of the SEI, researchers have explored several strategies such as the deposition of protective layers [17], material nanostructuration [14,18] and synthesis of Si composites [19,20,21,22]. Additionally, fluoroethylene carbonate (FEC) and vinylene carbonate (VC), which are electrolyte additives, have proved to be effective for achieving high-quality SEI [23,24,25], resulting in improved coulombic efficiencies and longer cycling life. However, while the use of FEC can be beneficial for the anode, depending on the amount added, it can be detrimental for cathode performance due to the formation of a thick layer on its surface, which can cause capacity fading and increase interfacial resistance [26].

In addition to the aforementioned issues, the combination of NCA cathodes and Si anodes has additional problems when LIBs are cycled at fast rates: cathode particle cracking, low active material utilization, side reactions of the electrode with the electrolyte and Li plating at the anode [27]. Achieving high C-rates in LIBs is a paramount target in the field of battery research. Although this is challenging, it is possible for cells to be assembled using very thin electrodes at the expense of the total energy density [28].

The aim of this work is the development of a Li-ion pouch cell with good operation stability at high C-rates (5C) through the integration of NCA and Si@G composite (containing 30 wt.% Si) as positive and negative electrodes, respectively, and the study of the possible degradation mechanisms of the cell at both electrode and electrolyte levels. For this purpose, (i) electrode materials are electrochemically characterized; (ii) the voltage window of the pouch cell is evaluated; (iii) the electrochemical performance of the pouch cell is determined; (iv) electrode materials are physicochemically evaluated after high C-rate cycling by X-ray diffraction, scanning electron microscopy and electrochemical impedance spectroscopy; and (v) electrolyte composition and stability are assessed by gas chromatography–mass spectrometry after cell cycling cell at high C-rates.

## 2. Results and Discussion

### 2.1. Materials Characterization

**Figure 1:**
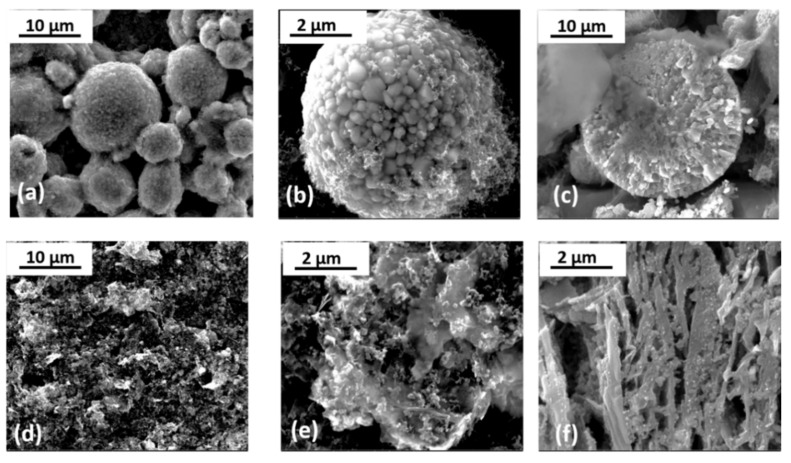
SEM micrographs of the pristine electrodes: NCA (**a**–**c**) and Si@G (**d**–**f**).

Figure 1 displays SEM images of the electrodes. It can be observed that the NCA pristine electrode consists of quasi-spherical smoothed secondary particles with diameters in the range of 3–15 µm (Figure 1a) formed by smaller primary particles of diameters below 1 µm (Figure 1b). It should be noted that the conductive additives are well distributed along the secondary particles, as can be seen in the same figure. The cross-section of a milled particle is shown in Figure 1c, where it can be observed that the secondary particles are well compacted. Regarding the anode, the pristine Si@G electrode shows a silicon-carbon matrix based on aggregates up to 10 µm of graphite particles decorated with Si nanoparticles (Figure 1d,e). Additionally, a more detailed view of the microstructure is depicted in Figure 1f, which corresponds to the cross-section of the anode showing parallel-oriented graphite flakes.

NCA and Si@G materials were also characterized by XRD (see diffractograms in Figure S1). The diffraction pattern registered for the NCA only shows diffraction peaks ascribed to the hexagonal LiNiO_2_ (COD1533770), discarding the presence of crystalline impurities. The pattern can be identified as an α-NaFeO_2_ structure with an *R*-3m space group. More insights about the structure are further discussed in Section 2.4.1. For the Si@G powder, characteristic diffraction peaks of hexagonal graphite (COD9012230) are present together with those of cubic silicon (COD9011998). A certain amount of silicon dioxide (marked with an asterisk) is also present in this sample, probably due to its formation during the synthesis procedure, which was carried out in air atmosphere.

### 2.2. Electrochemical Performance of Individual Electrodes: Half Cell

**Figure 2:**
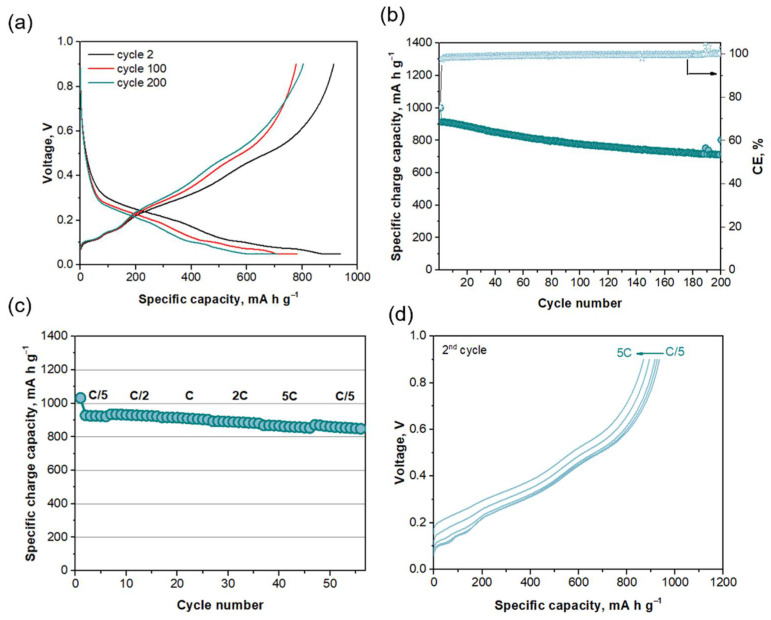
(**a**) Voltage–capacity curves of Cycles 2, 100 and 200 of Si@G electrode at C/5. (**b**) Specific capacity (mAh g^−1^; left axis) and coulombic efficiency (%, right axis) of Si@G electrode at C/5. (**c**) Specific capacity (mAh g^−1^) of Si@G electrode at different C-rates. (**d**) Charge voltage–capacity curves of the 2nd cycle of Si@G electrode at different C-rates. The term charge is referred to as the delithiation process.

The electrochemical performance of the Si@G composite was firstly evaluated in half-cell configuration. Figure 2a displays voltage–capacity curves registered for Cycles 2, 100 and 200, all exhibiting well-defined plateaus. The lower cut-off voltage was fixed at 0.05 V to limit the volume expansion experienced by silicon during prolonged cycling at low potentials that leads to poor stability [15]. During lithiation, below 0.2 V, the intercalation of lithium into graphite [29,30] and Si alloy formation take place. It was reported that, firstly, Li_~2_Si is formed at ca. 0.18 V, and after, the reaction continues to finally form Li_~3.5_Si at ca. 0.05 V [31]. As the polarization increases during cycling, the plateaus are shifted to lower potentials during lithiation. Figure 2b shows that the capacity after the activation cycle is 914 mAh g^−1^, 782 mAh g^−1^ after 100 cycles and 711 mAh g^−1^ at the end of the test (Cycle 200), which means that after 100 cycles, the capacity is over 85% of the initial cycle, and it is still over 78% at the end of the cycling test. The initial coulombic efficiency (ICE) is 75%, giving a value above 99% from the second cycle onwards.

The specific capacity of the Si@G electrode at different C-rates and the charge voltage–capacity curves are shown in Figure 2c,d. The Si@G anode shows good and stable performance at fast rates, with capacity values of 890 and 860 mAh g^−1^ at 2C and 5C (5 A g^−1^), respectively. It is noteworthy that our anode outperforms the capacity values reported previously [32,33,34,35] (see the information included in Table 1). The high and stable values of specific capacity obtained at high rates might be attributed to an efficient distribution of the particles achieved with the addition of isopropyl alcohol (IPA) during the synthesis, as well as the use of few-layer graphene (FLG) as conductive additive, which led to an optimal electrode microstructure able to buffer the expansion and contraction experienced by Si particles during cycling, as reported in our previous study [30]. Therefore, the electrochemical performance of the Si@G composite proves its suitability as anode for high-power Li-ion batteries. Its application in full-cell configuration is discussed in Section 3.3.

**Figure 3:**
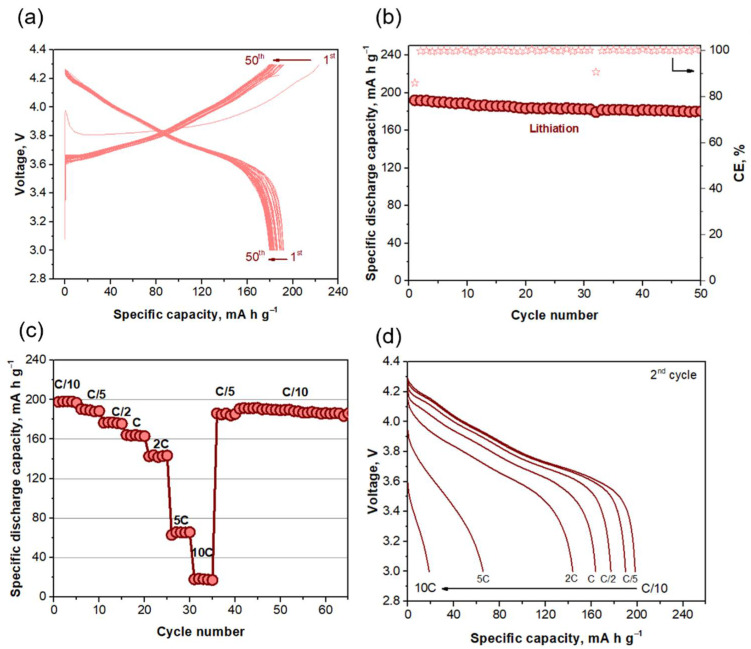
(**a**) Voltage–capacity curves (Cycles 1 to 50) of NCA electrode at C/5. (**b**) Specific capacity (mAh g^−1^; left axis) and coulombic efficiency (%, right axis) of NCA electrode at C/5. (**c**) Specific capacity (mAh g^−1^) of NCA electrode at different C-rates. (**d**) Discharge voltage–capacity curves of the 2nd cycle of NCA electrode at different C-rates. The term discharge is referred to as the lithiation process.

Regarding the electrochemical performance of the positive electrode, Figure 3a shows the voltage–capacity curves of the NCA electrode. During charge, the extraction of Li ions is associated with the phase transfer of four phases: hexagonal 1 (H1) to monoclinic (M), monoclinic to hexagonal 2 (H2) and hexagonal 2 to hexagonal 3 (H3) (see the differential capacity plot in Figure S2 for a clearer view), in agreement with Ref. [36]. These transitions are reversible during discharge (insertion of Li ions) and suffer a shift toward lower potentials during cycling. In addition, and only during the first delithiation, an overpotential that reaches a maximum of 3.99 V is noted, which drops to 3.8 V. Recent studies have attributed this overpotential to the decomposition of Li_2_CO_3_ formed by the reaction of NCA with air moisture and CO_2_ when the material is stored in ambient conditions and even during electrode preparation [37,38]. Capacity values remain stable at ca. 185 mAh g^−1^ during 50 cycles (Figure 3b), with an ICE of 86% that increases above 99.5% during cycling. Figure 3c depicts the capacity values obtained during the rate capability test, with values of 199, 190, 178, 165, 143, 65 and 18 mAh g^−1^ at C/10, C/5, C/2, C, 2C, 5C and 10C, respectively. The capacity is almost fully restored when cycling again at C/10 (190 mAh g^−1^) after fast rates, suggesting that the electrode has not deteriorated. The corresponding voltage–capacity curves are shown in Figure 3d.

### 2.3. Electrochemical Performance: Full Cell

#### 2.3.1. Evaluation of the Voltage Window

**Figure 4:**
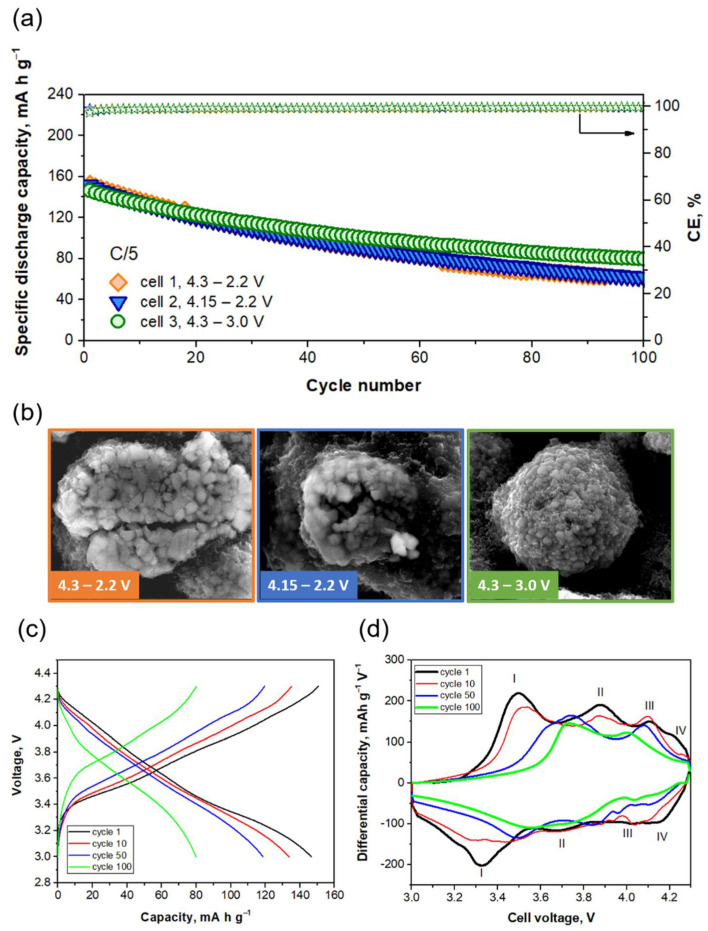
Evaluation of the voltage window. (**a**) Specific capacity (mAh g^−1^; left axis) and coulombic efficiency (%, right axis) of NCA/Si@G full cells (pouch cell configuration) cycled at C/5. (**b**) SEM images of the cycled NCA electrodes. (**c**) Voltage–capacity curves of Cell 3 cycled at C/5. (**d**) Differential capacity plot of Cycles 1, 10, 50 and 100 of Cell 3.

A study on the most appropriate voltage window (4.3–2.2 V, 4.15–2.2 V and 4.3–3.0 V) was carried out to achieve longer cyclability for the system at the pouch cell level. The cut-off voltage during charge was not higher than 4.3 V, as it is well known that high voltages and high temperatures contribute to the dissolution of cathode transition metals, leading to capacity fading and sometimes to cell failure [39]. The three cells present initial capacity values of 150 mAh g^−1^ (Figure 4a), with average specific capacities of 90, 95 and 105 mAh g^−1^ for Cells 1, 2 and 3, respectively. SEM images of the cycled positive electrodes (Figure 4b) depict flattened secondary particles and microcracks in the case of cells discharged at 2.2 V. Among several studies [40,41], there is no total agreement about the origin of microcracks in NCA particles. On the one hand, Watanabe and coworkers [42] claimed a strong correlation between microcrack formation and depth of discharge (DOD), concluding that the wider the DOD, the larger the volume changes in NCA particles and the more microcracks formed. On the other hand, a study from Dahn et al. [43] concluded that DOD was not exclusively the reason for microcrack formation. They demonstrated the importance of avoiding voltage regions of slow Li-ion diffusion during cycling, as the large Li concentration gradients formed in the particles under those conditions led to an increase in the internal strain and stress, resulting in particle fracturing. What seems clear is that the presence of microcracks produces an increase in the surface area of fractured NCA particles that are accordingly more exposed to electrolyte penetration. This can accelerate surface degradation and, eventually, give rise to NiO-like phase formation [41]. Formation of microcracks can also increase electrode resistance, as there is contact loss between particles. This could explain why capacity fading is more pronounced when 2.2 V is set as the lower cut-off voltage. In the case of the cell discharged at 3.0 V, the SEM image of the cycled positive electrode (Figure 4b) shows that secondary particles maintain their pristine spherical morphology. In addition, it can be seen that the conductive additive is still well distributed after 100 cycles, and the presence of microcracks cannot be detected, suggesting that this voltage window is the most suitable of the three studied not only for maintaining the electrode structure but also for keeping good contact between particles of active material and additives.

If we carefully evaluate the performance of Cell 3, during charge, we can distinguish four peaks ascribed to different processes (Figure 4c,d). The first charge is mainly dominated by Peak I at 3.5 V and Peak II at 3.9 V. The first is related to the alloy of Li with silicon nanoparticles forming Li_2_Si and the coexistence of H1 and M phases in NCA. Peak II is associated with Li intercalation on graphite and the coexistence of M and H2 phases in NCA. A minor contribution of Peaks III and IV is observed. Peak III at 4.1 V is related to a prolongation of Li intercalation on graphite, together with the formation of Li_3.5_Si alloy. Peak IV at 4.23 V is linked to the coexistence of H2 and H3 phases in NCA. During discharge, these processes are reversible, and deintercalation of Li from graphite leads to phase changes in NCA (H3 to H2 and H2 to M) in the region of 3.67–4.15. At 3.32 V, the dealloying reaction of Li_x_Si takes place. During cycling, Peaks I and II start to overlap in charge and discharge (Cycles 10 and 50) until they merge (Cycle 100), suggesting that chemical changes in electrode materials take place. Moreover, peak shifting (potential) is clear, and it is associated with an increase in internal resistance. This could explain the capacity fading observed in this cell during cycling.

#### 2.3.2. Rate Capability Test

**Figure 5:**
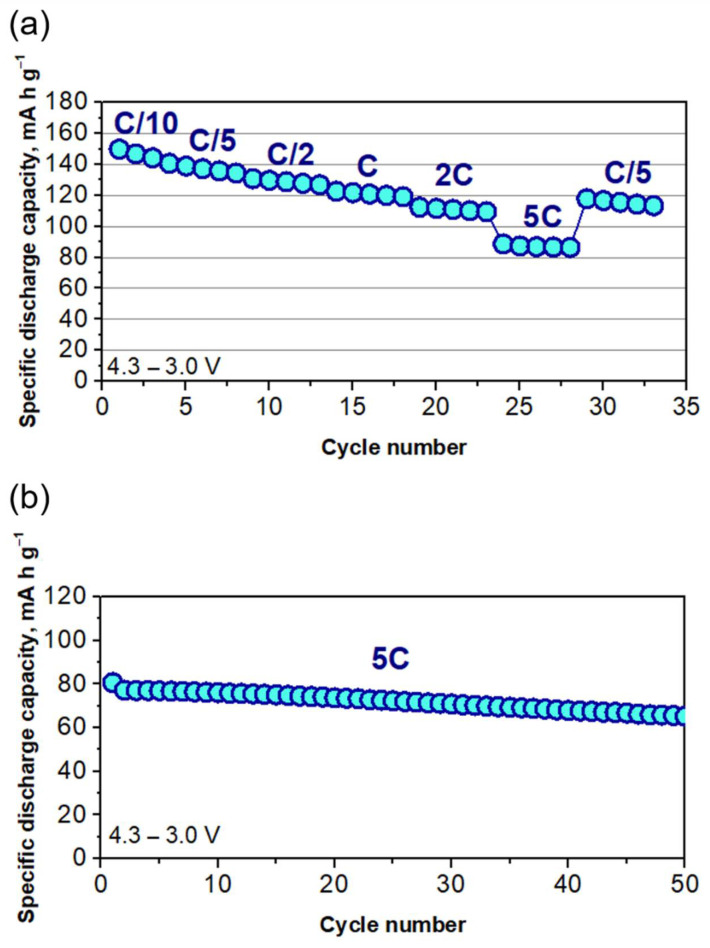
(**a**) Specific capacity (mAh g^−1^) of Cell 4 at different rates. (**b**) Specific capacity (mAh g^−1^) of Cell 4 cycled at 5C after 2 h of relaxation.

Regarding the behavior of the NCA/Si@G system at high C-rates, the pouch cell shows good stability and capacity retention, with capacity values of 147, 139, 130, 122, 112 and 87 mAh g^−1^ for C/10, C/5, C/2, C, 2C and 5C, respectively (Figure 5a). To the best of our knowledge, this is the first report on an NCA and silicon–graphite composite based pouch cell is reported at such a high cycling rate of 5C, achieving a capacity value of 87 mAh g^−1^, thus breaking the state of the art, currently reported 2C rate for an NCA/prelithiated silicon cell [44]. It is fair to highlight again the high wt.% of Si (30%) in the composite in comparison to that commonly used in industry (between 5 and 10 wt.%). In addition, the highly stable performance for 50 charge–discharge cycles at the 5C rate is shown in Figure 5b. For comparison, Table 2 shows the capacity values and cycling conditions of recent similar full cells [44,45,46].

As it can be seen, none of these studies reported electrode thickness values, which is known to be a crucial parameter mostly in cells cycled at high C-rates. One of the reasons for the good performance of the present cell at high C-rates could be ascribed to the fact that both positive and negative electrodes are rather thin: 45 µm for NCA and 30 µm for Si@G. Therefore, mass transport is less impeded, and rate capability is improved. This fact is in agreement with the work of Belharouak et al. [47], in which a specific capacity of 150 mAh g^−1^ was achieved at the 6C rate using thin NMC811 and graphite electrodes (49 µm and 58 µm, respectively). Figure S3 shows a differential capacity plot for Cycles 25 and 50, in which the main process taking place under this high rate (5C) is the phase change in NCA from H1 to H2, leading to the formation of Li_x_Si alloy. It can be seen that this process is reversible in both cycles. For Cycle 50, the peak shift (potential) is only 0.02 V, suggesting that there is no increase in internal resistance.

### 2.4. Characterization after Cycling

#### 2.4.1. Electrode Morphology and Structure

**Figure 6:**
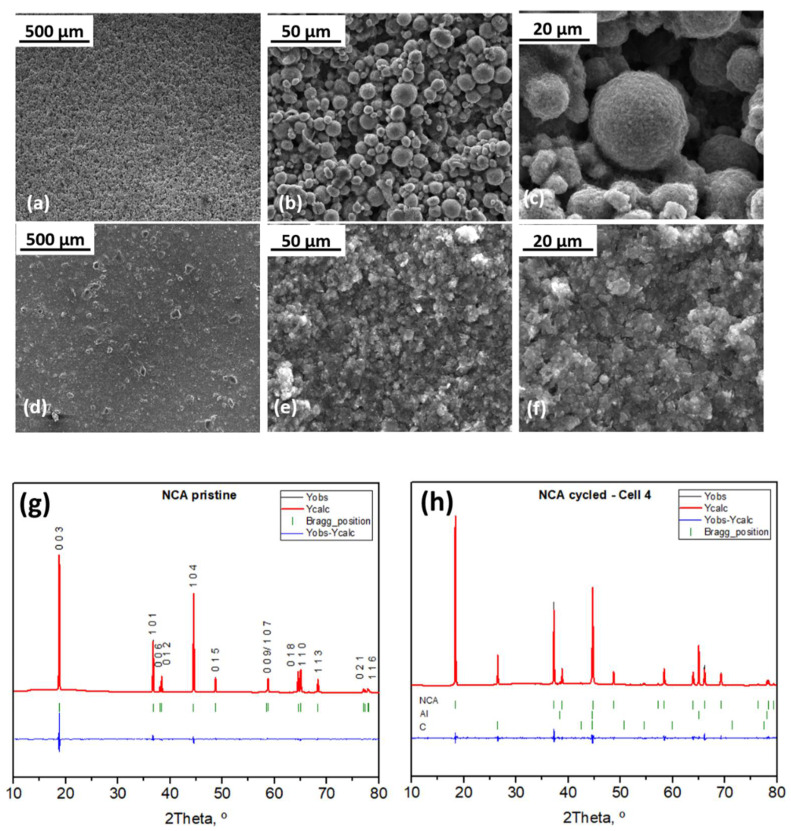
SEM images of the cycled electrodes: NCA (**a**–**c**) and Si@G (**d**–**f**). Rietveld refinements of the XRD data for (**g**) pristine NCA electrode and (**h**) NCA electrode cycled at 5C rate.

It is worth highlighting that, even after being cycled at 5C for 50 cycles, the pouch cell does not swell. Electrodes are not detached from the current collector, and they do not show any evidence of lithium plating nor material loss (see photographs in Figure S4). In addition, the separator (not shown) did not suffer any mechanical stress, resulting undamaged, and there is no material attached to it. Focusing on the electrode surfaces, SEM images of the NCA electrode show no cracks along the electrode (Figure 6a). The quasi-spherical morphology of the NCA particles is maintained, being quite similar to that in the pristine electrode, and there is no evidence of microcracks in the particles (Figure 6b,c). In addition, the conductive additives remain well distributed surrounding and covering the NCA spheres, as shown in Figure 6c. A homogeneous distribution of the NCA electrode elements (O, Ni, Co, Al and C) is shown in Figure S5. Particle morphology is also preserved at the anode (Figure 6d,f). In this case, although there are no cracks at the electrode, the presence of agglomerates can be observed, probably due to the volume changes undergone by the silicon. Even at high rates of 5C, Li plating does not occur, so it can be assumed that the N/P ratio remains above 1. Transition metals (migration from the cathode) were not detected in the anode through elemental analysis (data not shown). These results suggest that strains and stress during cycling do not significantly affect the electrodes. The absence of particle cracking contributes, in part, to better stability during cycling, as there is no material loss.

With respect to the structure, and as mentioned in Section 2.1, the NCA has a hexagonal unit cell with space group *R*-3m. Its lattice parameters are a = 2.865(2) Å and c = 14.184(5) Å (Table S1). Li and metal ions occupy 3a and 3b sites, whereas 6c sites are occupied by oxygen ions in a close-packed oxygen array with an ABCAB sequence. Rietveld refinement of the diffractograms of the cycled cells at the state of discharge was carried out in order to determine if cycling conditions affected the structure of the electrodes (Figure 6g,h). After cycling at the 5C rate, diffraction peaks arising from NCA can be noticed. Diffraction peaks at 26.5 and at 65° correspond to C from the conductive additive and to Al from the current collector, respectively. The diffractogram registered for the cycled NCA electrode does not show any additional diffraction peak nor exhibit significant peak shifting. The refined lattice parameters (Table S1) indicate a slight contraction of the unit cell in the *a*-axis, while there is an expansion in the *c*-axis. In addition, the volume of the cell is slightly reduced, suggesting that the NCA structure is preserved, and does not undergo major changes under the high-rate cycling regime imposed. Moreover, considering that the 003/104 intensity ratio is important to estimate the degree of Li/M disorder, the cycled NCA electrode presents values higher than typical values of 1.2–1.3, which indicates a low degree of cation mixing [48]. Thus, it can be stated that transition metal ions are not present in the Li plane, and consequently, Li diffusion is not hampered.

#### 2.4.2. Electrolyte Composition

**Figure 7:**
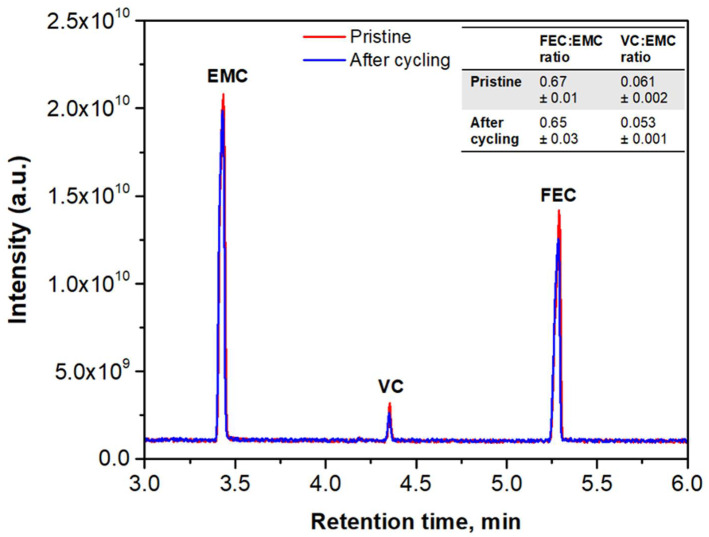
GC–MS chromatogram of pristine electrolyte (red), which did not undergo any electrochemical cycling, and the aged electrolyte, which was cycled at the 5C rate (blue). Three main components were identified: EMC (RT = 3.42 min), VC (RT = 4.35 min) and FEC (RT = 5.26 min).

In order to gain further insight into the stability of the cell, GC–MS measurements were performed. This is a semiquantitative method that has been proved to be useful to assess electrolyte composition and identify any variation after electrochemical cycling [49]. In Figure 7, the main peaks identified by GC–MS for the pristine electrolyte and cycled electrolyte are shown: EMC, with a retention time of 3.42 min; VC, with a retention time of 4.35 min; and FEC, with a retention time of 5.26 min. Mass spectra of these compounds are shown in Figure S7. While peak intensities slightly fluctuated, relative mass abundances remained similar in both cases. In fact, the FEC–EMC ratio was 0.65 ± 0.03, which was not significantly different from the pristine electrolyte (0.67 ± 0.01), while the VC–EMC ratio had a value of 0.053 ± 0.001 (slightly lower than the pristine electrolyte: 0.061 ± 0.002). Consequently, none of the additives suffered any significant change after SEI formation and fast cycling.

Other studies have oppositely shown FEC degradation in Si–C composite electrodes as the number of cycles is increased, leading to a rapid capacity drop of the cell [50,51]. It is known that in Si-based anodes, FEC is involved in the formation and evolution of the SEI [23,52,53]. One cause of FEC consumption in these anodes is related to a continuous SEI formation that occurs due to the volume expansion undergone by Si particles during cycling, causing breaking of the SEI. Based on our previous results [30], our anode has an optimal microstructure that is able to mitigate Si volume changes during cycling. Similarly, no significant capacity fading appeared after 50 charge–discharge cycles (see Figure 5b). Therefore, we can assume that constant surface film formation is prevented, as is FEC consumption. On the other hand, the fact that the VC–EMC ratio in the cycled cell is almost similar to that presented in the case of the pristine electrolyte is in agreement with other studies that report on the suppression of the decomposition of other electrolyte components to a minimum due to the presence of FEC in the electrolyte [50,54]. Hence, a high-rate cycling regime is possible with almost no degradation of the electrolyte.

#### 2.4.3. Impedance of the Full Cell

**Figure 8:**
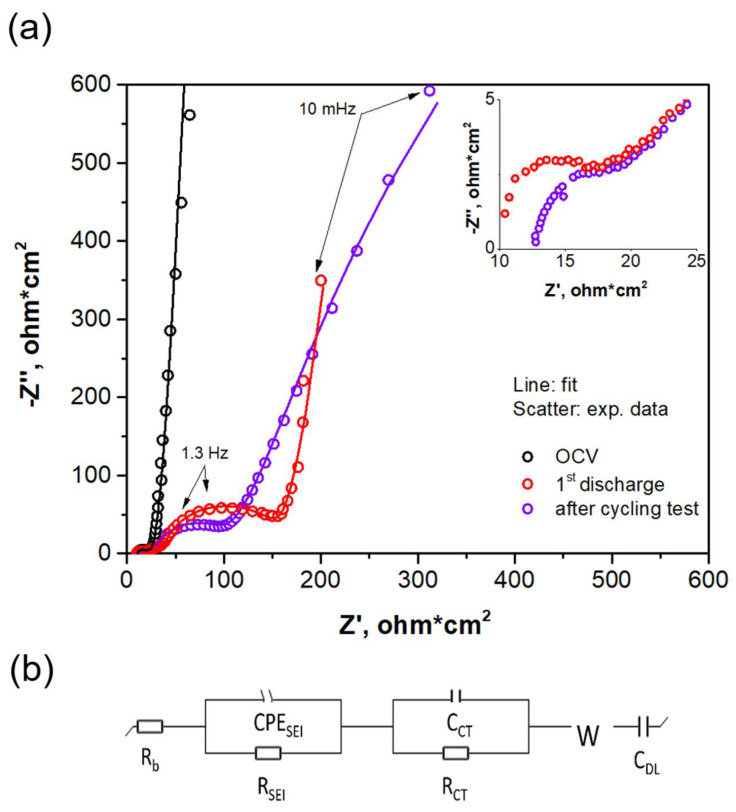
(**a**) Nyquist plots (lines: fit; scatter: experimental data) of the NCA/Si@G full cell (pouch cell configuration) at OCV state after the first discharge and after the cycling test at 5C rate. Voltage window: 4.3–3.0 V. The inset shows a magnification of the high-frequency zone. (**b**) Equivalent circuit used for fitting the spectra.

Figure 8a shows Nyquist plots of the full cell before and after cycling in the frequency range of 500 kHz–10 mHz. For the fitting of the spectra, the selected equivalent circuit can be found in Figure 8b. The circuit is composed of three types of resistances: *R_b_*, which is the bulk resistance associated with components (electrode materials, current collector, separator and electrolyte); *R_SEI_*, related to interface resistance of the solid electrolyte interphase films; and *R_ct_*, associated with the charge transfer resistance. All the data have been normalized with regard to the electrode area. At open-circuit voltage, the first semicircle observed in the high-frequency zone is attributed to *R_b_*, as there is no contribution of *R_SEI_* due to the uncycled state. In the cycled cells, the semicircles at high and midfrequency zones are attributed to *R_SEI_* and *R_ct_*, respectively. For a clearer view of *R_SEI_*, the high-frequency region has been expanded and is shown in the inset of Figure 8a. The segment in the region of mid to low frequency found in the spectra has a profile of almost vertical lines, which represent a combination of resistive and capacitive behavior, and it was fitted with a combination of *C_DL_* (capacitor element related to double-layer capacitance) with *W* (Warburg element related to ion diffusion).

With the purpose of elucidating the contribution of each electrode to the full-cell impedance, Nyquist plots for symmetric cells are shown in Figure S6. It can be assumed that *R_ct_* in the full cell is attributed to the resistance offered by the NCA-positive electrode to Li insertion, while the segment present in the mid- to low-frequency region, associated with capacitive behavior as mentioned above, is totally attributed to the Si@G-negative electrode. In addition, in the case of the negative electrode, a smooth semicircle is observed at high frequency, suggesting that a better-defined SEI exists in the Si@G anode.

The total resistance of the full cell (*R_b_* + *R_SEI_* + *R_ct_*) after the first discharge is 135 Ω·cm^2^ and 89.1 Ω·cm^2^ in the last cycle. It is known that *R_ct_* increases during cycling [55,56,57] due to the depth of discharge, thickening of the SEI and selected working potentials, among others. However, at the end of cycling at the 5C rate, *R_ct_* is lower (53.60 Ω·cm^2^) than in the first discharge (103.17 Ω·cm^2^). Thus, it can be stated that kinetics of charge transfer to electrode material is improved due to a stabilized surface of the NCA-positive electrode caused by stable solid electrolyte interphase on the cathode, in agreement with the work of Liu and Manthiram [58], who reported an improved charge transfer kinetics of LiMn_1.5_Ni_0.42_Zn_0.08_O_4_ due to stable surface chemistries that give optimum SEI layers. Moreover, Wise et al. [59] used an Al_2_O_3_ coating on an NMC cathode to successfully mitigate the strong interactions of the material with the electrolyte at high voltages, achieving a stable electrode surface that resulted in smaller resistances. Other researchers have observed that electrolyte additives play an important role in maintaining stable films on the electrode particle surface by inducing suppression of the impedance growth [60]. Therefore, at the full-cell level, not only the smaller *R_ct_* arisen from the NCA electrode but also the capacitive behavior provided by the Si@G electrode support the cycling stability results at the 5C rate, indicating that cycling the NCA/Si@G pouch cell at a high-rate cycling regime does not cause large increases in the total cell resistance, nor does it slow down Li-ion diffusion processes.

## 3. Materials and Methods

### 3.1. Anode Preparation

For the preparation of the Si@G composite, silicon nanoparticles (Alfa Aesar, Haverhill, MA, USA), graphite (Imerys, Paris, France) and IPA (Scharlab, Barcelona, Spain) were mixed in a planetary mill (Pulverisette 5, Fritsch, Idar-Oberstein, Germany) at 400 rpm for 2 h. Additional details of the synthesis are reported in our previous work [30]. The Si@G composite was used as active material and mixed with FLG, supplied by the University of Cambridge (UCAM, Cambridge, UK), and lab-made lithium polyacrylic acid (LiPAA), according to the following formulation: 85 wt.% active material, 10 wt.% conductive additive and 5 wt.% binder. The content of Si in the active material was 30 wt.%. Materials were mixed using a magnetic stirrer at a speed of 1000 rpm. The obtained slurry was casted onto copper foil (Schlenk) and dried at 40 °C overnight without vacuum to prevent detachment of the material from the current collector. The final thickness was ca. 30 µm. The electrodes were punched according to an electrode area of 1.13 cm^2^ and 21.84 cm^2^ for coin cell and pouch cell configuration, respectively. Finally, the electrodes were dried at 120 °C under vacuum overnight immediately before their use in cells.

### 3.2. Cathode Preparation

Commercial NCA powder (Targray), used as active material, was mixed with the binder PVDF (Solvay SOLEF ^®^ 5130) and conductive additives C65 and graphite (Imerys), according to the following formulation: 80 wt.% NCA, 10 wt.% PVDF and 5 wt.% C65 + 5 wt.% graphite. Materials were mixed in a dissolver (Dispermat ^®^ CV3-PLUS) with a total processing time of 7 h and a maximum speed of 2000 rpm. The slurry was then casted onto aluminum foil (Hohsen Corp.) and vacuum dried at 80 °C for 2 h. Then, the laminates were calendered, achieving a final thickness of 45 µm. The electrodes were punched according to an electrode area of 1.13 cm^2^ and 20.25 cm^2^ for coin cell and pouch cell configuration, respectively. Finally, the electrodes were dried at 120 °C under vacuum overnight immediately before their use in cells.

### 3.3. Electrochemical Testing

Half cells (CR2032-type coin cells) were assembled inside a glove box under an argon atmosphere. The anode or the cathode was used as positive electrode, a disc of metallic lithium as negative electrode and a Whatman glass fiber disc as separator. The electrolyte solution used was 1 M lithium hexafluorophosphate (Li[PF_6_]) in fluoroethylene carbonate (FEC) and ethyl methyl carbonate (EMC) (FEC:EMC = 3:7) with 2 wt.% vinylene carbonate (VC). Monolayer pouch cells were assembled inside a dry room with a dew point of −60 °C and a temperature of 22 °C. In this case, a Celgard 2325 membrane was used as separator.

The electrochemical properties of the Si@G and NCA electrodes were tested by galvanostatic cycling in half cells (coin cell type). On the anode side, stability tests were performed at symmetric discharge/charge C/5 rate in a potential range of 0.05−0.9 V. Furthermore, to evaluate the rate capability, symmetric discharge/charge C-rates were varied from C/5 to 5C. Both stability and rate capability tests include an activation cycle performed at C/10 (potential range of 0.005–0.9 V) and a constant current constant voltage (CCCV) step at C/20 at the end of each lithiation. On the cathode side, NCA was charged and discharged at a C/5 rate between 3.0 and 4.3 V. The same voltage window was selected to perform the rate capability test, varying C-rates (symmetric charge/discharge) from C/10 to 10C. Regarding the electrochemical performance of the full cell, NCA/Si@G pouch cells were cycled at symmetric charge/discharge C/5 rate in the voltage ranges of 4.3–2.2 V (Cell 1), 4.15–2.2 V (Cell 2) and 4.3–3.0 V (Cell 3) using a negative (N) to positive (P) ratio of 1.3 (assuming 1259.7 mAh g^−1^ as the theoretical capacity of the Si@G anode and 185 mAh g^−1^ as the practical capacity of the NCA cathode). Finally, an NCA/Si@G pouch cell was cycled in the potential window of 4.3–3.0 V at different C-rates (symmetric charge/discharge), ranging from C/10 to 5C (Cell 4). For both half- and full-cell configurations, the terms charge and discharge are referred to as delithiation and lithiation processes, respectively. Specific capacity values (mAh g^−1^) are attributed to grams of Si@G active material for the Si@G half cell, grams of NCA active material for the NCA half cell and grams of NCA active material for the NCA/Si@G full cell.

### 3.4. Physicochemical Characterization of Electrodes and Electrolyte

Structural analysis of both active powdered materials was performed through XRD using a Bruker D8 Discover diffractometer using Cu-Kα radiation. The XRD patterns were refined by Rietveld methods using FullProf software in order to estimate lattice parameters, with iterative refinement of background, peak shape function coefficients and lattice parameters, until convergence was reached. For determining the morphology of the powders and electrodes, SEM measurements were carried out in a FEG Quanta 200 from Thermo Fisher operated at 20 kV. Additionally, in order to measure the resistance of the electrodes, EIS of the full cells (pouch cell configuration) at room temperature was performed, both at OCV and at discharged state (all the cells were stopped at 3.0 V). Measurements were performed in a VMP-3 apparatus (Biologic ^®^) in the frequency range of 500 kHz–10 mHz, with an amplitude of 20 mV and 5 measures per frequency. EC-Lab software was used for fitting the spectra. Finally, the electrolyte composition was measured by GC–MS, a gas chromatograph (Clarus ^®^ 590, Perkin Elmer, Waltham, MA, USA) coupled to a mass spectrometer (Clarus ^®^ SQ 8 T, Perkin Elmer, Waltham, MA, USA) in order to determine if any degradation of the same occurred. The sample (0.5 µL) was subjected to the following oven temperature program: starting with 45 °C for 2.50 min, the temperature was then increased up to 290 °C for 5 min (ramp of 40 °C/min). Data acquisition was conducted after a solvent delay of 2.22 min in the mass range of m/z = 20–300. Compounds were identified by their retention times and by matching their mass profile with the NIST MS Search Library.

## 4. Conclusions

A Li-ion pouch cell with good operation stability at high C-rates was successfully developed through the integration of NCA as cathode and Si@G composite (with 30% Si in its structure) as anode. On one hand, the use of silicon allows achieving the same or even higher capacity values with less electrode mass compared to a common graphite anode. On the other hand, the anode microstructure allows achieving fast rates, as volume changes undergone by silicon particles are mitigated. In consequence, the pouch cell presented here shows reversible capacity values of 87 mAh g^−1^ (in the rate capability test) and ca. 70 mAh g^−1^ (in the stability test for 50 cycles) at a rate of 5C, reported for the first time in the literature for the NCA/Si@G full cell.

In addition, the evaluation of the aged components by several characterization techniques confirms the good stability of the electrode materials and electrolyte, which were not degraded according to the following evidence: (i) particle morphology is maintained as in the raw materials in both the cathode and anode, and no migration of transition metals is detected. In addition, electrode delamination and cracking phenomena were not observed. (ii) Parameters of the unit cell did not undergo significant changes, maintaining their pristine structure. (iii) Li-ion diffusion processes were not hampered at fast rates, as confirmed by EIS and diffractogram refinement analysis. (iv) Electrolyte degradation is almost negligible, discarding the formation of undesired products on the electrode surface. Last but not least, no plating was detected, which is very important for safety issues.

In summary, the novelty of this study relies on the suitability of the electrode combination used, which is able to demonstrate very good electrochemical behavior in terms of power capability, without evidence of damage to the battery. It is fair to highlight that these results are achieved without applying any anode prelithiation strategy, which could lead to even higher capacity values. Similarly, using higher electrode loadings could lead to higher stable capacity values at fast rates. Hence, the NCA/Si@G Li-ion battery presented here is positioned as a promising candidate for mitigating the problem of high-power applications.

## Figures and Tables

**Table 1:** Comparison of recent similar half cells using a Si-based anode.

Reference	% of Si in the Anode	Voltage Window	Capacity, mAhg^−1^	Current Rate
Stability Tests				
This work	30 wt.%	0.05–0.9 V	844 (Cycle 50); 711 (Cycle 200)	C/5
Ma et al. [32]	17 wt.%	0.005–1.0 V	790 (Cycle 50, end of the test)	C/2
Hsu et al. [33]	30 wt.%	0.05–1.5 V	590 (Cycle 50); 600 (Cycle 200)	C/5
Rate Capability Tests				
This work	30 wt.%	0.05–0.9 V	860	5C (5 A g^−^^1^)
Huang et al. [34]	Not reported	0.01–1.5 V	700	1.2 A g^−^^1^
Sui et al. [35]	12.8 wt.%	0.01–1.5 V	458	2 A g^−^^1^

**Table 2:** Comparison of recent similar full cells. Cell configuration, voltage window, capacity values, electrode thickness and loading are shown.

Reference	Battery	Configuration	Voltage Window	Capacity, mAh/g	Electrode Thickness	Electrode Loading
This work	Si–graphite composite anode and NCA cathode	Pouch cell	4.3–3.0 V	130 @ C/2112 @ 2C87 @ 5C	Cathode: 45 µm Anode: 30 µm	Cathode: 5 mg/cm^2^ Anode: 1 mg/cm^2^
Wagner et al. [44]	Prelithiated Si anode and NCA cathode	Coin cell	4.4–2.2 V	150 @ C/2110 @ 2C	Not reported	Cathode: 3.5 mg/cm^2^ Anode: 0.6 mg/cm^2^
Winter et al. [45]	Si–graphite composite anode and NCA cathode	Coin cell	4.4–3.0 V	145 @ C/2	Not reported	Cathode: 5–5.5 mg/cm^2^ Anode: not reported
Eom et al. [46]	Si–graphene anode and NCA cathode	Pouch cell	4.2–2.75 V	ca. 125 @ C/2	Not reported	Cathode: not reported Anode: 1.65 mg/cm^2^

## Supplementary Materials

The following are available online at https://www.mdpi.com/article/10.3390/ijms221910331/s1, Figure S1: Diffractograms of the powder materials. Figure S2: Differential capacity plot of NCA. Figure S3: Differential capacity plot of NCA/Si@G pouch cell. Figure S4: Photographs of pouch cell electrodes. Figure S5: Energy-dispersive X-ray (EDX) mapping of the NCA electrode recovered from the pouch cell. Figure S6: Nyquist plots of symmetric cells. Figure S7: Mass profiles of (a) EMC, (b) VC and (c) FEC. Table S1: Results from Rietveld refinement.
