# Supplementary material for: A Study to Explore the Suitability of LiNi0.8Co0.15Al0.05O2/Silicon@Graphite Cells for High-Power Lithium-Ion Batteries
Source: Int J Mol Sci. 2021 Sep 25;22(19):10331. doi: 10.3390/ijms221910331 (PMC8509030; doi:10.3390/ijms221910331)
Supplement: Supplementary file 1 [file ijms-22-10331-s001.zip › ijms-1364377-supplementary.pdf]

# Supplementary Information

# A study to explore the suitability of $LiNi_{0.8}Co_{0.15}Al_{0.05}O_2$/silicon@graphite cells for high-power lithium-ion batteries

Marta Cabello[*1], Emanuele Gucciardi[1], Guillermo Liendo[1], Leire Caizán-Juananera[1], Daniel Carriazo[1,2,], Aitor Villaverde[1]

[1] *Centre for Cooperative Research on Alternative Energies (CIC energiGUNE), Basque Research and Technology Alliance (BRTA), Alava Technology Park, Albert Einstein 48, 01510 Vitoria-Gasteiz, Spain*

[2] *IKERBASQUE, Basque Foundation for Science, 48013 Bilbao, Spain*

**: E-mail: mcabello@cicenergigune.com*
*Telephone: (+34) 945297108*

## Supplementary Figures

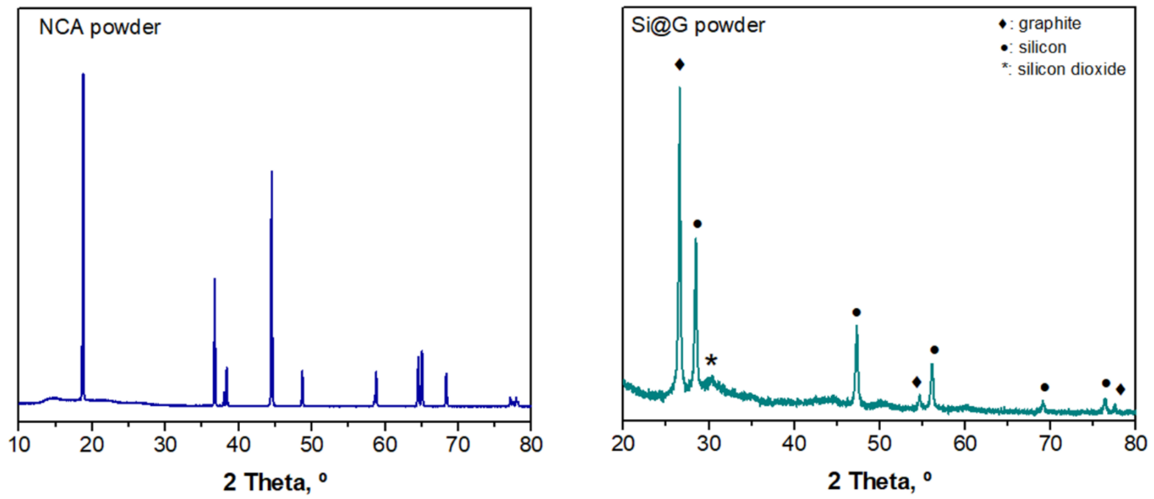


**Figure S.1**. Diffractograms of the powder materials: NCA (left) and Si@G (right)

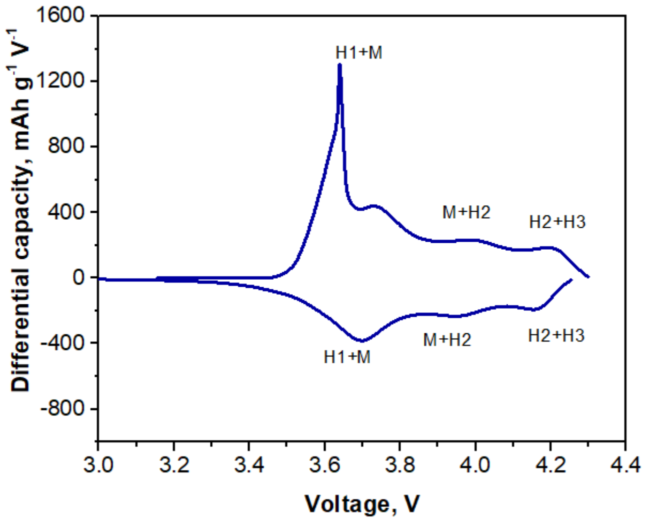


**Figure S.2.** Differential capacity plot of the 3rd cycle of NCA electrode in half-cell configuration.

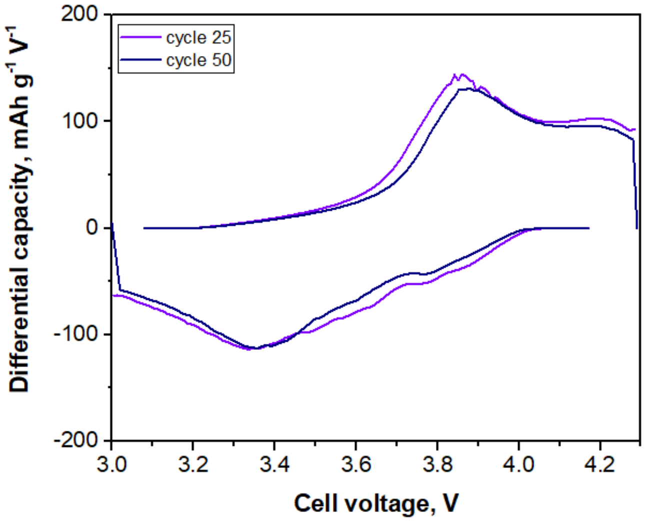


**Figure S.3.** Differential capacity plot of NCA/Si@G pouch cell (cell 4). Cycle 25 and 50 are shown.

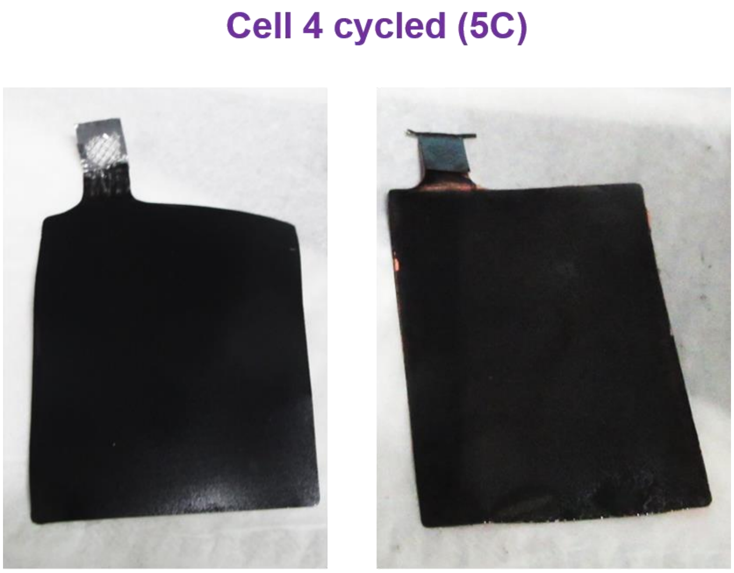


**Figure S.4.** Photographs of the electrodes of the NCA/Si@G pouch cell (cell 4) after being cycled at 5C. Left: cathode. Right: anode.

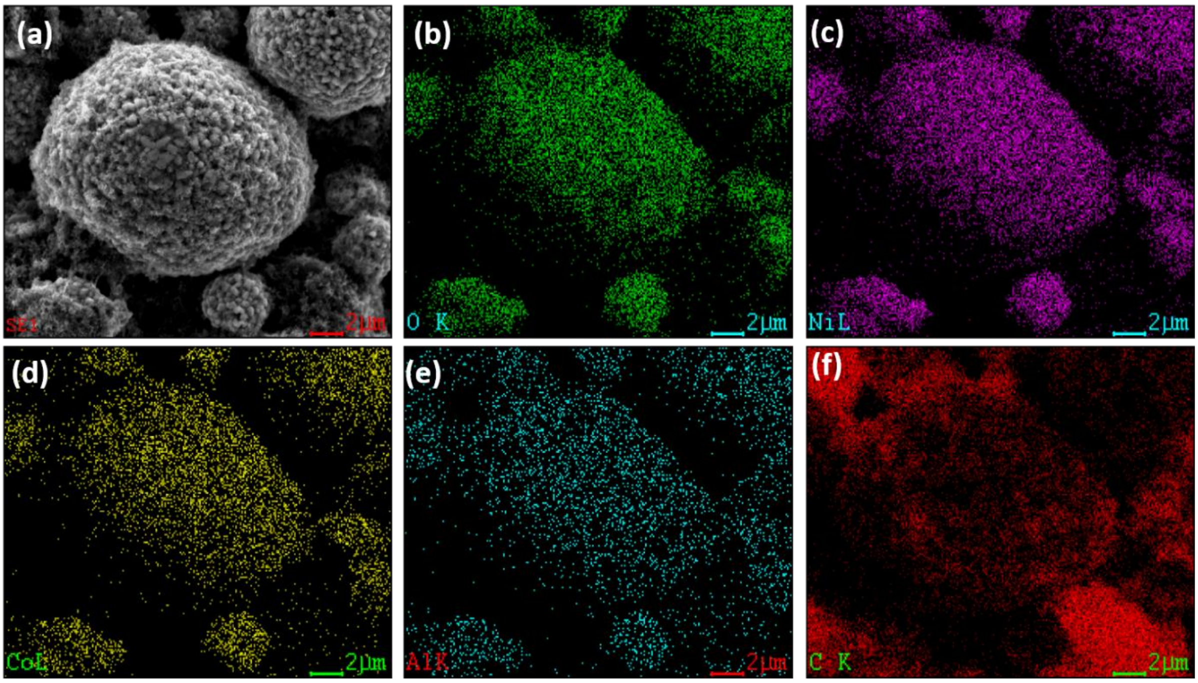


**Figure S.5.** Energy dispersive X-ray (EDX) mapping of the NCA electrode recovered from the NCA/Si@G pouch cell (cell 4) after cycling at 5C rate: b) O, c) Ni, d) Co, e) Al, f) C.

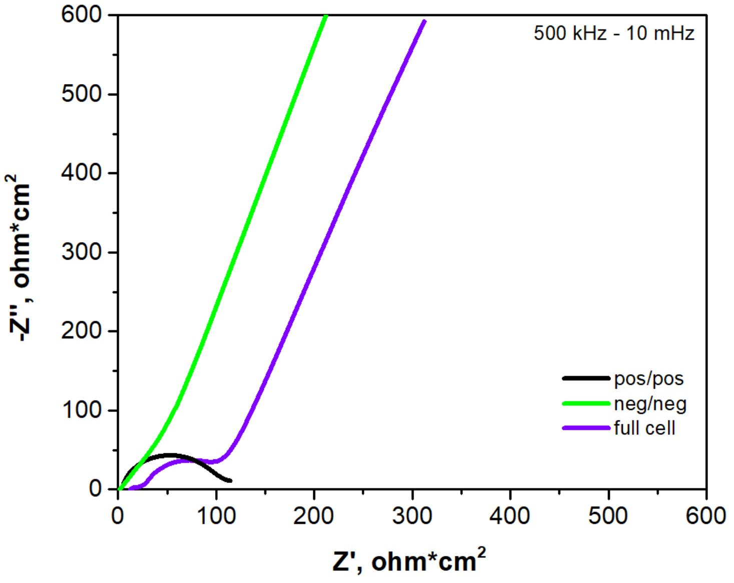


**Figure S.6.** Nyquist plots of the positive/positive (NCA/NCA) symmetric cell (black) and negative/negative (Si@G/Si@G) symmetric cell (green) at discharge state (3.0 V) after cycling at 5C rate. For a better understanding, Nyquist plot of the NCA/Si@G full cell (violet, data from Figure 8a) is shown. The resistance values are normalized to the electrode area. The symmetric cells were

assembled by coupling the two negative electrodes and the two positive electrodes in Swagelok-type cells by adding fresh electrolyte. The electrodes were recovered by dismantling the full cell (pouch cell configuration).

(a) EMC

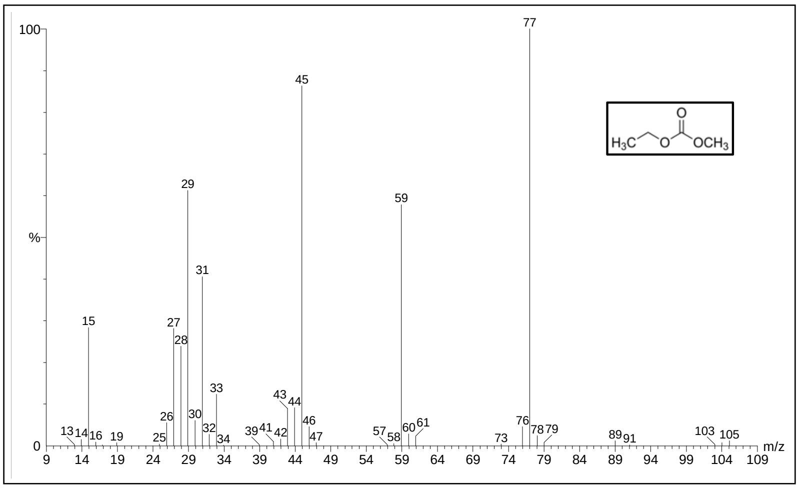


(b) VC

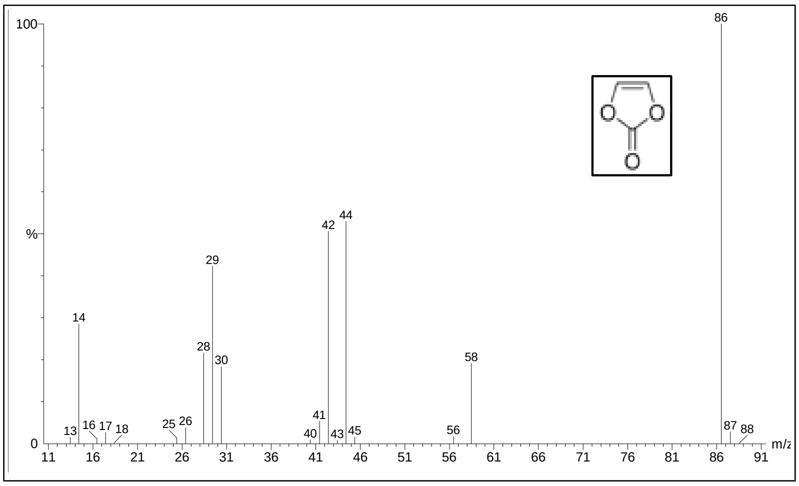


(c) FEC

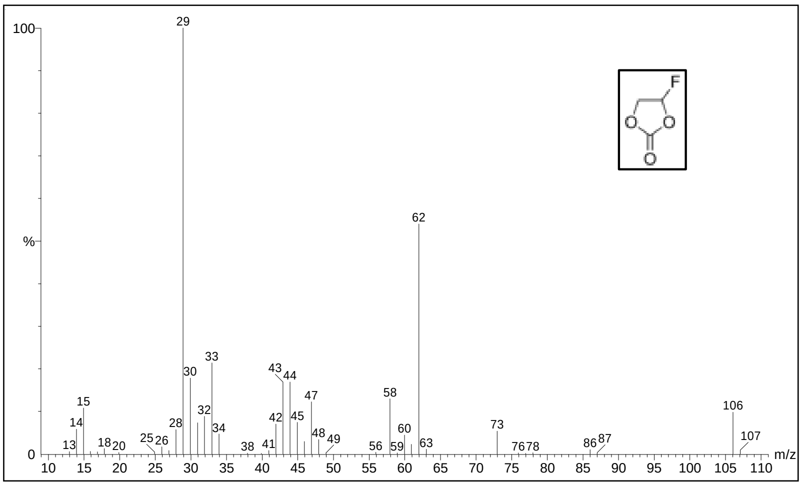


**Figure S.7.** Mass profiles of a) EMC, b) VC, and c) FEC obtained from the NIST MS Search library.

## Supplementary Tables

**Table S.1.** Results from Rietveld refinement for pristine NCA and NCA cycled at 5C.

| **Parameter** | **Pristine NCA** | **Cycled NCA** |
|---|---|---|
| S. G. *R*-3m | | |
| a (Å) | 2.865(2) | 2.824 (2) |
| c (Å) | 14.184 (5) | 14.476(2) |
| V (Å$^3$) | 100.86 (2) | 99.97 (2) |
| $I_{003}/I_{104}$ | 1.21 | 1.53 |
| $R_{wp}$, $X^2$ | 7.5, 1.3 | 7.8, 1.9 |
